# Diagnostic accuracy and added value of dynamic chest radiography in detecting pulmonary embolism: A retrospective study

**DOI:** 10.1016/j.ejro.2024.100602

**Published:** 2024-10-05

**Authors:** Yuzo Yamasaki, Kazuya Hosokawa, Takeshi Kamitani, Kohtaro Abe, Koji Sagiyama, Takuya Hino, Megumi Ikeda, Shunsuke Nishimura, Hiroyuki Toyoda, Shohei Moriyama, Masateru Kawakubo, Noritsugu Matsutani, Hidetake Yabuuchi, Kousei Ishigami

**Affiliations:** a Department of Clinical Radiology, Graduate School of Medical Sciences, Kyushu University, Japan; b Department of Cardiovascular Medicine, Graduate School of Medical Sciences, Kyushu University, Japan; c Department of Hematology, Oncology & Cardiovascular Medicine, Graduate School of Medical Sciences, Kyushu University, Japan; d Department of Health Sciences, Graduate School of Medical Sciences, Kyushu University, Japan; e Healthcare Business Headquarters, KONICA MINOLTA, INC., Japan

**Keywords:** Dynamic chest radiography, Pulmonary embolism, Diagnostic accuracy, Contrast-enhanced computed tomography

## Abstract

**Purpose:**

This study aimed to assess the diagnostic performance of dynamic chest radiography (DCR) and investigate its added value to chest radiography (CR) in detecting pulmonary embolism (PE).

**Methods:**

Of 775 patients who underwent CR and DCR in our hospital between June 2020 and August 2022, individuals who also underwent contrast-enhanced CT (CECT) of the chest within 72 h were included in this study. PE or non-PE diagnosis was confirmed by CECT and the subsequent clinical course. The enrolled patients were randomized into two groups. Six observers, including two thoracic radiologists, two cardiologists, and two radiology residents, interpreted each chest radiograph with and without DCR using a crossover design with a washout period. Diagnostic performance was compared between CR with and without DCR in the standing and supine positions.

**Results:**

Sixty patients (15 PE, 45 non-PE) were retrospectively enrolled. The addition of DCR to CR significantly improved the sensitivity, specificity, accuracy, and area under the curve (AUC) in the standing (35.6–70.0 % [*P* < 0.0001], 84.8–93.3 % [*P* = 0.0010], 72.5–87.5 % [*P* < 0.0001], and 0.66–0.85 [*P* < 0.0001], respectively) and supine (33.3–65.6 % [*P* < 0.0001], 78.5–92.2 % [*P* < 0.0001], 67.2–85.6 % [*P* < 0.0001], and 0.62–0.80 [*P* = 0.0002], respectively) positions for PE detection. No significant differences were found between the AUC values of DCR with CR in the standing and supine positions (P = 0.11) or among radiologists, cardiologists, and radiology residents (P = 0.14–0.68).

**Conclusions:**

Incorporating DCR with CR demonstrated moderate sensitivity, high specificity, and high accuracy in detecting PE, all of which were significantly higher than those achieved with CR alone, regardless of scan position, observer expertise, or experience.

## Highlights

• DCR showed moderate sensitivity, high specificity, and high accuracy in detecting PE.
• The addition of DCR increased the diagnostic ability of observers for PE detection.
• These findings highlight the potential of DCR as a valuable diagnostic tool for PE.

## Graphical Abstract

**Figure:**
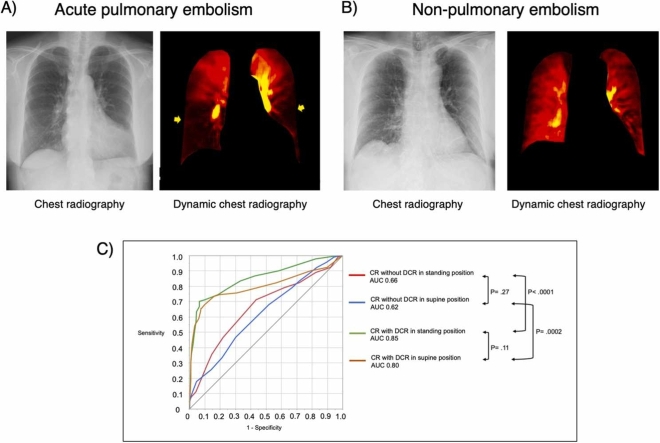

A) Images of chest radiography (left) and dynamic chest radiography (right) in a patient with acute pulmonary embolism. Large wedge-shaped perfusion defects are observed in bilateral lungs (arrows).
B) Images of chest radiography (left) and dynamic chest radiography (right) in a patient without pulmonary embolism.
C) Receiver operating characteristic curves for CR without DCR in the standing position, CR without DCR in the supine position, CR with DCR in the standing position, and CR with DCR in the supine position. Significant differences are observed between CR without DCR and CR with DCR both in standing and supine positions. CR, chest radiography; DCR, dynamic chest radiography.

## 1 Introduction

Pulmonary embolism (PE) is a prevalent life-threatening condition. In the United States, the reported annual incidence of acute PE ranges from 39 to 115 cases per 100,000 people, with an age-adjusted mortality rate of 14.4 % for PE-related deaths [1], [2]. It is the third leading cause of cardiovascular death in the United States after myocardial infarction and stroke [3]. Early diagnosis is critical for improving clinical outcomes [4], [5], which underscores the importance of a readily available diagnostic method without contraindications.

Chest radiography (CR) and echocardiography are part of the initial workup for patients with suspected PE to rule out other causes of chest pain. Occasionally, typical chest radiographic findings can aid in the diagnosis of PE; however, the sensitivity of these signs is low, and a diagnosis based on CR is usually difficult [6]. Dynamic chest radiography (DCR), a novel imaging technique that uses a conventional X-ray system with a flat-panel detector and pulsed X-ray generator to assess pulmonary perfusion, has recently been introduced and garnered increasing popularity. This non-invasive procedure can be performed without contrast media in a general radiology room, together with CR. The total patient dose can be less than the International Atomic Energy Agency-recommended dose limit for two projections (posteroanterior and lateral views) of chest radiographs [7], [8]. DCR reveals wedge-shaped perfusion defects in acute pulmonary thromboembolism [9], [10], [11], [12]. Additionally, it has been demonstrated to be as effective as ventilation/perfusion scintigraphy in detecting chronic thromboembolic pulmonary hypertension (CTEPH) among patients with pulmonary hypertension [13], [14]. Thus, DCR is expected to increase the detectability of PE in the general population when combined with CR. DCR reveals findings similar to those of ventilation/perfusion scintigraphy [15], [16], [17]. In the last few decades, scintigraphy has been largely replaced with contrast-enhanced CT (CECT) as the preferred diagnostic tool for detecting PE, particularly in acute scenarios. Currently, CECT has high diagnostic accuracy for detecting PE and is recommended as the first option for suspected PE. However, the use of CECT is limited in cases of contrast allergies, renal dysfunction, pregnancy, or the absence of a CT machine. In contrast, DCR offers a non-invasive assessment of pulmonary perfusion without the need for contrast. Additionally, DCR can be performed in a general X-ray examination room and provides prompt results, making it a potential option for patients with suspected PE. However, while previous studies have focused on PE detection using ventilation/perfusion scintigraphy as the standard [13], [15], data on the diagnostic performance of DCR for PE using CECT as a reference standard are still lacking. Furthermore, since the DCR system can obtain both chest X-ray and DCR images, the added value of DCR over chest X-ray alone should be evaluated for clinical application.

An additional unique feature of DCR is its positional flexibility, allowing it to be performed in both standing and supine positions. The suitable position for PE detection has not yet been investigated.

Therefore, this study aimed to evaluate the diagnostic accuracy and added value of DCR for the detection of PE compared to CR alone in the standing and supine positions.

## 2 Methods

This diagnostic study was approved by the institutional review board of our hospital (No. 2022-180), and the requirement for informed consent was waived owing to the retrospective nature of the study. This study was performed in accordance with the Declaration of Helsinki. One author (N.M.) was an employee of Konica Minolta, Inc. and provided technical support in the development of image analysis software but was not involved in the study design or data interpretation. The institutional authors, who are not Konica Minolta, Inc. employees, controlled the data.

### 2.1 Study population

From June 2020 to August 2022, 775 consecutive patients at our hospital underwent both CR and DCR. Among these patients, individuals aged 18 years or older who also underwent CECT of the chest within 72 h were selected and screened for potential inclusion in the study. The exclusion criteria were (a) history of chest surgery, (b) absence of standing or supine positional DCR, (c) final diagnosis of CTEPH, and (d) poor image quality due to motion artifacts. Diagnoses of PE and non-PE were confirmed using CECT. In addition, non-PE was reconfirmed by at least 1 month of follow-up of clinical records to rule out misinterpretation of CECT. We excluded patients with CTEPH from the study cohort to focus on acute or subacute PE cases.

### 2.2 Dynamic chest radiography

All patients underwent DCR with 7–10 s of inspiratory breath-holding using a flat-panel detector (AeroDR fine, KONICAMINOLTA) and a pulsed X-ray generator (RADspeed Pro, SHIMADZU), together with CR. Imaging was performed in standing and supine positions. Detailed scan parameters were set based on the body mass indices of the patients following a previous study [18] (Supplementary Table 1). Dynamic perfusion images and lung perfusion maps were generated from sequential DCR images using a workstation (KINOSIS V1.20R00, KONICAMINOLTA, Inc.) as previously reported [13], [19]. Temporal changes in pixel values in sequential images acquired during breath holding were calculated and analyzed. The temporal changes in the pixel values from the end-diastolic phase were color-coded and visualized as dynamic perfusion images. The maximum-intensity-projection image in the lung, which was determined by semiautomatic contouring based on an edge detection algorithm, was created as a lung perfusion map and used for the evaluation in conjunction with the dynamic perfusion images.

### 2.3 Observer study

A fully crossed, multiple-reader, and multiple-case study was conducted in two sessions to avoid potential bias in the sequential reading design [20]. A total of 60 patients were randomized into two groups (Group A [n = 30] and Group B [n = 30]). In the first session, each observer assessed Group A without DCR and Group B with DCR. In the second session, each observer assessed Group A with DCR and Group B without DCR. The first and second sessions were scheduled with a minimum interval of 2 weeks between them, and the order of radiographs was randomized and differed from the previous session to reduce recall bias. Six observers who were not involved in the image review or reference standard settings independently graded the images. The readers were blinded to the clinical information, imaging reports, and numbers of patients with and without PE included in the study. Two observers (T.H. and T.K.) were board-certified thoracic radiologists with 9 and 23 years of experience, two (M.K. and S.M.) were board-certified cardiologists with 7 and 9 years of experience, and two (S.N. and H.T.) were radiology residents. The observers were instructed on the typical signs of PE in CR (the Westermark sign, Hampton hump, knuckle sign, and Fleischner sign) and how to read the DCR. DCR images were evaluated following the previously reported diagnostic flow [13], [19]. Lung perfusion maps and dynamic perfusion images were used to assess the DCR. Accompanying CR was used to reference the lung border and assess lung abnormalities (mass/nodules, infiltration, reticular shadows, bulla, pneumothorax, and pleural effusion). A well-circumscribed wedge-shaped abnormality on dynamic perfusion images (or a lung perfusion map) with normal lung findings on CR was defined as an embolic-type perfusion defect. The presence of at least one embolic-type defect led to the final diagnosis of PE. All observers reported their confidence levels in the presence of PE by assigning numbers ranging from 0 (lowest confidence level) to 100 (highest confidence level). The images were displayed without annotations or DICOM tags during the observer test. Prior to the first session, all observers were individually trained using 20 CRs and DCRs, including 10 PE and 10 non-PE cases that were excluded from the observer test. The observers were presented with CR and DCR images and were trained on how to differentiate PE and non-PE cases with reference to the corresponding CT images.

### 2.4 Statistical analysis

Statistical analyses were performed using JMP Pro version 16.0.0 (SAS Institute Inc., NC, USA) and GraphPad Prism version 8.4.3 (GraphPad Software, Inc., CA, USA). The normality of distributions was assessed using the Shapiro–Wilk test. A two-tailed Student's *t*-test (for normally distributed variables) or Mann–Whitney *U* test (for non-parametric variables) was conducted to compare the clinical data between the PE and non-PE groups. The statistical significance of the difference in diagnostic performance (sensitivity, specificity, and accuracy) between CR with and without DCR and between the standing and supine positions was assessed using the McNemar test. Receiver operating characteristic (ROC) curve analysis was used to calculate the area under the curve (AUC), and the difference in AUCs between CR with and without DCR and that between the standing and supine positions were tested using the DeLong method. The reading times of CR with DCR among radiologists, cardiologists, and radiology residents were compared using a one-way repeated analysis of variance with Tukey's post-hoc test for multiple comparisons. All data are presented as mean ± standard deviation. Statistical significance was set at *P* < 0.05.

## 3 Results

### 3.1 Patient characteristics

Between June 2020 and August 2022, a total of 775 patients underwent both CR and DCR. Among them, 694 were excluded from the study due to a lack of CECT performed within 72 h. Furthermore, 16 patients were excluded based on the final diagnosis of CTEPH, and an additional five patients were excluded due to a previous history of thoracic surgery. Finally, the analysis included 60 patients, consisting of 15 individuals with PE and 45 without PE, as confirmed by CECT and subsequent clinical follow-up. The patient demographics are shown in Table 1. The mean intervals between DCR and CECT were 19.0 ± 18.1 h and 25.2 ± 15.7 h in PE and non-PE groups, respectively. The entrance surface doses for the DCR were 1.0 ± 0.2 mGy and 1.6 ± 0.5 mGy in the standing and supine positions, respectively.

**Table 1:** Patient characteristics.

	PE group	Non-PE group	P value
Number	15	45	-
Age	62.9 ± 13.3	69.3 ± 12.3	.09
Sex (male/female)	10/5	14/31	.015
BSA (m^2^)	1.6 ± 0.2	1.6 ± 0.2	.56
Interval between DCR and CECT (h)	19.0 ± 18.1	25.2 ± 15.7	.21
Comorbidities			
・Hypertension ・Diabetes ・Hyperlipidemia ・COPD ・Interstitial pneumonia ・Dialysis ・Chronic heart disease ・Malignancy	7 2 5 0 0 0 2 3 1:Pancreatic cancer 1:Esophageal cancer 1:Endometrial cancer	17 7 6 2 1 1 5 36 12:Renal cell carcinoma 12:Esophageal cancer 5:Colon cancer 5:Gastric cancer 1:Cardiac sarcoma 1:Gall bladder cancer	-

*Note: Data are presented as the mean ± SD or number of patients. Two-tailed Student's *t*-test (normally distributed variables) or the Mann–Whitney *U* test (non-parametric variables) was performed to compare the clinical data between the PE and non-PE groups. Categorical variables were compared using the Fisher’s exact test.

PE, pulmonary embolism; BSA: Body surface area; DCR, dynamic chest radiography; CECT, contrast-enhanced chest computed tomography; COPD, chronic obstructive pulmonary disease.

### 3.2 Observer performance with and without DCR

Table 2, Table 3 present the performances of the observers with and without DCR images in the standing and supine positions, respectively. The addition of DCR in the standing and supine positions improved the pooled observer sensitivity (35.6–70.0 % [*P* < 0.0001] and 33.3–65.6 % [*P* < 0.0001], respectively), specificity (84.8–93.3 % [*P* = 0.0010] and 78.5–92.2 % [*P* < 0.0001], respectively), accuracy (72.5–87.5 % [*P* < 0.0001] and 67.2–85.6 % [*P* < 0.0001], respectively) and AUC (0.66–0.85 [*P* < 0.0001] and 0.62–0.80 [*P* = 0.0002], respectively) (Table 3). The pooled observer diagnostic performances of DCR with CR were high, with accuracies > 85 % and AUCs of ≥ 0.8 observed in both standing and supine positions.

**Table 2:** Performance of observers without and with DCR in the standing position.

	AUC	Sensitivity (%)	Specificity (%)	PPV (%)	NPV (%)	Accuracy (%)	Reading time (s)
**Performance of observers without DCR**							
Pooled observers	**.66**	**35.6 (32/90) [27.4–43.9]**	**84.8 (229/270) [82.1–87.6]**	**43.8 (32/73) [33.7–54.1]**	**79.8 (229/287) [77.2–82.4]**	**72.5 (261/360) [68.4–76.6]**	**32 ± 13**
Observer 1 (Radiologist)	.68	33.3 (5/15) [14.1–54.3]	84.4 (38/45) [78.0–91.4]	41.7 (5/12) [17.7–67.8]	79.2 (38/48) [73.2–85.7]	71.7 (43/60) [62.1–82.1]	36 ± 13
Observer 2 (Radiologist)	.49	13.3 (2/15) [2.4–29.8]	91.1 (41/45) [87.5–96.6]	33.3 (2/6) [6.1–74.4]	75.9 (41/54) [72.9–80.5]	71.7 (43/60) [66.2–79.9]	21 ± 6
Observer 3 (Cardiologist)	.74	33.3 (5/15) [14.2–53.2]	86.7 (39/45) [80.3–93.3]	45.5 (5/11) [19.4–72.6]	79.6 (39/49) [73.7–85.7]	73.3 (44/60) [63.8–83.3]	38 ± 4
Observer 4 (Cardiologist)	.68	66.7 (10/15) [41.8–85.9]	68.9 (31/45) [60.6–75.3]	41.7 (10/24) [26.1–53.7]	86.1 (31/36) [75.7–94.1]	68.3 (41/60) [55.9–78.0]	23 ± 8
Observer 5 (Radiology resident)	.70	20.0 (3/15) [5.7–36.6]	91.1 (41/45) [86.4–96.6]	42.9 (3/7) [12.3–78.4]	77.4 (41/53) [73.3–82.1]	73.3 (44/60) [66.2–81.6]	49 ± 7
Observer 6 (Radiology resident)	.78	46.7 (7/15) [24.7–66.2]	86.7 (39/45) [79.4–93.2]	53.8 (7/13) [28.5–76.4]	83.0 (39/47) [76.0–89.2]	76.7 (46/60) [65.7–86.4]	24 ± 7

**Performance of observers with DCR**							
Pooled observers	**.85**	**70.0 (63/90) [62.2–76.3]**	**93.3 (252/270) [90.7–95.4]**	**77.8 (63/81) [69.2–84.7]**	**90.3 (252/279) [87.8–92.3]**	**87.5 (315/360) [83.6–90.6]**	**45 ± 21**
Observer 1 (Radiologist)	.89	53.3 (8/15) [31.4–67.5]	93.3 (42/45) [86.0–98.0]	72.7 (8/11) [42.8–92.0]	85.7 (42/49) [79.0–90.0]	83.3 (50/60) [72.4–90.4]	33 ± 7
Observer 2 (Radiologist)	.78	40.0 (6/15) [21.9–40.0]	100 (45/45) [94.0–100]	100 (6/6) [54.7–100]	83.3 (45/54) [78.3–83.3]	85.0 (51/60) [75.9–85.0]	31 ± 7
Observer 3 (Cardiologist)	.94	80.0 (12/15) [56.4–93.9]	88.9 (40/45) [81.0–93.5]	70.6 (12/17) [49.8–82.9]	93.0 (40/43) [84.8–97.9]	86.7 (52/60) [74.9–93.6]	71 ± 11
Observer 4 (Cardiologist)	.84	73.3 (11/15) [51.1–84.1]	95.6 (43/45) [88.2–99.1]	84.6 (11/13) [59.0–97.0]	91.5 (43/47) [84.4–94.9]	90.0 (54/60) [78.9–95.4]	31 ± 10
Observer 5 (Radiology resident)	.94	100 (15/15) [78.8–100]	86.7 (39/45) [79.6–86.7]	71.4 (15/21) [56.3–71.4]	100 (39/39) [91.9–100]	90.0 (54/60) [79.4–90.0]	69 ± 17
Observer 6 (Radiology resident)	.92	73.3 (11/15) [51.1–84.1]	95.6 (43/45) [88.2–99.1]	84.6 (11/13) [59.0–97.0]	91.5 (43/47) [84.4–94.9]	90.0 (54/60) [78.9–95.4]	32 ± 4

**P value (without DCR vs with DCR)**							
Pooled observers	**< .0001**	**< .0001**	**.0010**	**-**	**-**	**< .0001**	**< .0001**
Observer 1 (Radiologist)	.013	.26	.21	-	-	.090	.14
Observer 2 (Radiologist)	.027	.10	.041	-	-	.011	< .0001
Observer 3 (Cardiologist)	.0022	.020	.71	-	-	.046	< .0001
Observer 4 (Cardiologist)	.14	.71	.0013	-	-	.0046	< .0001
Observer 5 (Radiology resident)	.0023	<.0001	.48	-	-	.025	< .0001
Observer 6 (Radiology resident)	.082	.10	.10	-	-	.021	< .0001

*Note: Data in parentheses are 95 % CIs. Data in brackets are raw data. Reading times are presented as means standard deviations. DCR, dynamic chest radiography; AUC, area under the receiver operating characteristic curve; PPV, positive predictive value; NPV, negative predictive value.

**Table 3:** Performance of observers without and with DCR in the supine position.

	AUC	Sensitivity (%)	Specificity (%)	PPV (%)	NPV (%)	Accuracy (%)	Reading time (s)
**Performance of observers without DCR**							
Pooled observers	**.62**	**33.3 (30/90) [25.0–42.1]**	**78.5 (212/270) [75.8–81.4]**	**34.1 (30/88) [25.6–43.1]**	**77.9 (212/272) [75.2–80.8]**	**67.2 (242/360) [63.1–71.6]**	**38 ± 15**
Observer 1 (Radiologist)	.54	40.0 (6/15) [18.7–63.8]	66.7 (30/45) [59.6–74.6]	28.6 (6/21) [13.4–45.6]	76.9 (30/39) [68.7–86.1]	60.0 (36/60) [49.4–71.9]	32 ± 10
Observer 2 (Radiologist)	.70	26.7 (4/15) [9.9–40.9]	93.3 (42/45) [87.8–98.1]	57.1 (4/7) [21.3–87.6]	79.2 (42/53) [74.5–83.3]	76.7 (46/60) [68.3–83.8]	29 ± 8
Observer 3 (Cardiologist)	.60	26.7 (4/15) [9.4–50.0]	75.6 (34/45) [69.8–83.3]	26.7 (4/15) [9.4–50.0]	75.6 (34/45) [69.8–83.3]	63.3 (38/60) [54.7–75.0]	42 ± 10
Observer 4 (Cardiologist)	.55	53.3 (8/15) [29.6–76.0]	53.3 (24/45) [45.4–60.9]	27.6 (8/29) [15.3–39.3]	77.4 (24/31) [65.9–88.4]	53.3 (32/60) [41.5–64.6]	36 ± 15
Observer 5 (Radiology resident)	.62	0 (0/15) [0–6.3]	97.8 (44/45) [97.8–99.9]	0 (0/1) [0–94.5]	74.6 (44/59) [74.6–76.2]	73.3 (44/60) [73.3–76.5]	53 ± 13
Observer 6 (Radiology resident)	.75	53.3 (8/15) [30.3–73.3]	84.4 (38/45) [76.8–91.1]	53.3 (8/15) [30.3–73.3]	84.4 (38/45) [76.8–91.1]	76.7 (46/60) [65.2–86.7]	37 ± 17

**Performance of observers with DCR**							
Pooled observers	**.80**	**65.6 (59/90) [57.5–72.2]**	**92.2 (249/270) [89.5–94.5]**	**73.8 (59/80) [64.7–81.3]**	**88.9 (249/280) [86.3–91.1]**	**85.6 (308/360) [81.5–88.9]**	**45 ± 16**
Observer 1 (Radiologist)	.83	53.3 (8/15) [32.7–59.6]	97.8 (44/45) [90.9–99.9]	88.9 (8/9) [54.5–99.4]	86.3 (44/51) [80.2–88.1]	86.7 (52/60) [76.4–89.8]	33 ± 8
Observer 2 (Radiologist)	.79	53.3 (8/15) [34.0–53.3]	100 (45/45) [93.5–100]	100 (8/8) [63.7–100]	86.5 (45/52) [81.0–86.5]	88.3 (53/60) [78.7–88.3]	37 ± 6
Observer 3 (Cardiologist)	.74	66.7 (10/15) [42.8–84.4]	86.7 (39/45) [78.7–92.6]	62.5 (10/16) [40.1–79.1]	88.6 (39/44) [80.5–94.7]	81.7 (49/60) [69.7–90.5]	62 ± 13
Observer 4 (Cardiologist)	.90	86.7 (13/15) [62.9–97.5]	84.4 (38/45) [76.5–88.1]	65.0 (13/20) [47.2–73.2]	95.0 (38/40) [86.1–99.1]	85.0 (51/60) [73.1–90.4]	36 ± 13
Observer 5 (Radiology resident)	.82	66.7 (10/15) [44.5–77.5]	95.6 (43/45) [88.2–99.2]	83.3 (10/12) [55.7–96.9]	89.6 (43/48) [82.7–93.0]	88.3 (53/60) [77.3–93.7]	60 ± 10
Observer 6 (Radiology resident)	.76	66.7 (10/15) [43.1–83.6]	88.9 (40/45) [81.0–94.5]	66.7 (10/15) [43.1–83.6]	88.9 (40/45) [81.0–94.5]	83.3 (50/60) [71.5–91.8]	42 ± 16

**P value (without DCR vs with DCR)**							
Pooled observers	**.0002**	**< .0001**	**<.0001**	**-**	**-**	**< .0001**	**< .0001**
Observer 1 (Radiologist)	.023	.41	.0002	-	-	.0003	.092
Observer 2 (Radiologist)	.35	.16	.24	-	-	.035	<.0001
Observer 3 (Cardiologist)	.31	.058	.096	-	-	.0012	<.0001
Observer 4 (Cardiologist)	.0015	.096	.0010	-	-	.0003	.15
Observer 5 (Radiology resident)	.076	.0001	.032	-	-	.0067	< .0001
Observer 6 (Radiology resident)	.94	.48	.56	-	-	.37	.11

*Note: Data in parentheses are 95 % CIs. Data in brackets are raw data. Reading times are presented as means standard deviations. DCR, dynamic chest radiography; AUC, area under the receiver operating characteristic curve; PPV, positive predictive value; NPV, negative predictive value.

**Fig. 1:**
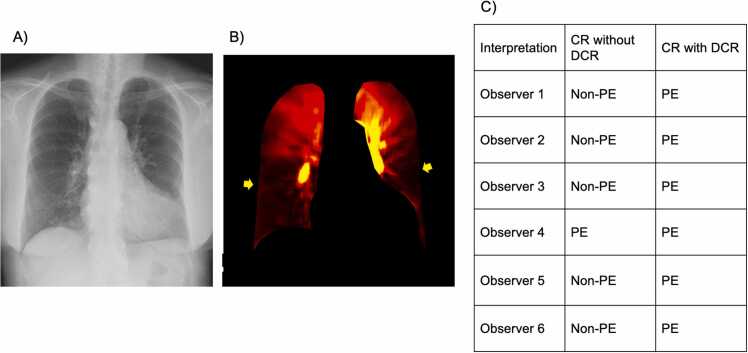
CR (A) and DCR (B) images of a 67-year-old woman with acute pulmonary thromboembolism captured in the standing position. Perfusion defects due to thromoboembolism (B, arrows) are illustrated in the bilateral lungs in the DCR image, with normal findings on the corresponding area in the CR image. All observers except for observer 4 interpreted the CR image as negative for PE. By adding the DCR images, five of the six observers changed their interpretations correctly (C). * CR, chest radiography; DCR, dynamic chest radiography; PE, pulmonary embolism.

Representative PE and non-PE cases with observer interpretations are shown in Fig. 1 (and Supplementary Video 1) and 2 (and Supplementary Video 2), respectively.

**Fig. 2:**
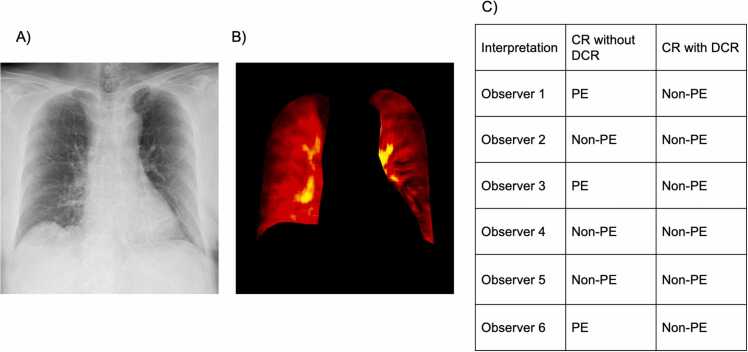
CR (A) and DCR (B) images of a 50-year-old man without pulmonary embolism captured in the supine position. Interpretations of the CR images varied among the observers, with observers 1, 3, and 6 identifying PE and observers 2, 4, and 5 interpreting it as non-PE. After adding the DCR image, three of the six observers correctly changed their interpretations (C). * CR, chest radiography; DCR, dynamic chest radiography; PE, pulmonary embolism.

### 3.3 Comparison between the diagnostic performance in the standing and supine positions

**Fig. 3:**
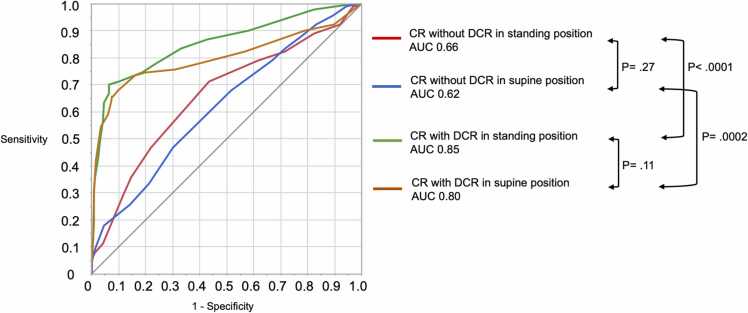
Receiver operating characteristic curves for CR without DCR in the standing position, CR without DCR in the supine position, CR with DCR in the standing position, and CR with DCR in the supine position. CR, chest radiography; DCR, dynamic chest radiography.

The addition of DCR to CR significantly improved the diagnostic performance in both the standing and supine positions (standing, *P* < 0.0001; supine, *P* < 0.0001) (Table 2, Table 3, and Fig. 3). The performance of CR with DCR in the standing position was higher than that in the supine position; however, the difference was not significant (AUC 0.85 vs. 0.80, *P* = 0.11) (Fig. 3).

### 3.4 Comparison of diagnostic performances among radiologists, cardiologists, and radiology residents

**Fig. 4:**
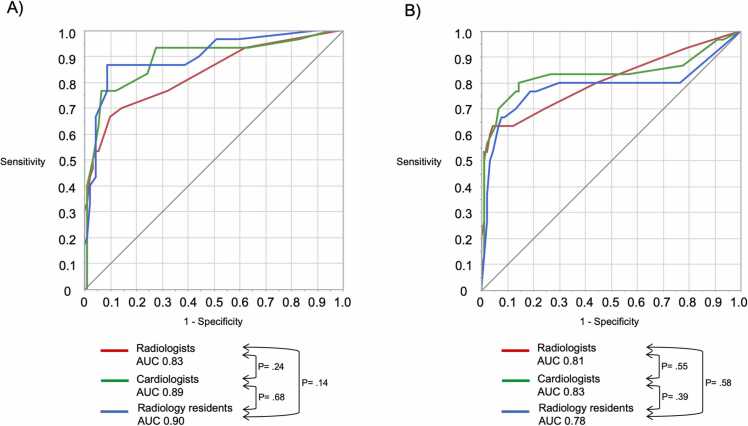
Comparison of the areas under the curves for the diagnostic performance of radiologists using dynamic chest radiography in the standing (A) and supine (B) positions with that of cardiologists and radiology residents.

Subgroup analysis of observer performance according to expertise and experience is shown in Table 4, Table 5. The addition of DCR to CR improved the diagnostic performances in all groups for the standing (accuracy: radiologists [71.7–84.2 %], *P* = 0.0039; cardiologists [70.8–88.3 %], *P* = 0.0006; radiology residents [75.0–90.0 %], *P* = 0.0015; AUC: radiologists [0.55–0.83], *P* = 0.0002; cardiologists [0.69–0.89], *P* = 0.0028; radiology residents [0.75–0.90], *P* = 0.0011) and supine positions (accuracy: radiologists [68.3–87.5], *P* < 0.0001; cardiologists [58.3–83.3], *P* < 0.0001; radiology residents [75.0–85.8], *P* = 0.020; AUC: radiologists [0.61–0.81], *P* = 0.013; cardiologists [0.57–0.83], *P* = 0.0024; radiology residents [0.69–0.78], *P* = 0.28). Except for the AUC of the radiology resident in the supine position, all observed differences were statistically significant. The utilization of DCR resulted in high AUC values across all groups. There were no significant differences observed between the AUC values of the radiologists, cardiologists, and radiology residents in both the standing (0.83 vs. 0.89 vs. 0.90, *P* = 0.14–0.68) and supine (0.81 vs. 0.83 vs. 0.78, *P* = 0.39–0.58) positions (Fig. 4). Observer performances of DCR for PE detection in standing and supine positions are summarized in Supplementary Table 2.

**Table 4:** Subgroup analysis of observer performance according to the observer’s expertise and experience in the standing position.

	AUC	Sensitivity (%)	Specificity (%)	PPV (%)	NPV (%)	Accuracy (%)	Reading time (s)
**Performance of observers without DCR**							
Radiologists (n = 2)	.55	23.3 (7/30) [11.5–36.9]	87.8 (79/90) [83.8–92.3]	38.9 (11/18) [19.1–61.6]	77.5 (79/102) [74.0–81.5]	71.7 (86/120) [65.6–78.5]	28 ± 13
Cardiologists (n = 2)	.69	50.0 (15/30) [33.9–65.4]	77.8 (70/90) [72.4–82.9]	42.9 (15/35) [29.0–56.1]	82.4 (70/85) [76.7–87.8]	70.8 (85/120) [62.8–78.5]	31 ± 10
Radiology residents (n = 2)	.75	33.3 (10/30) [19.6–46.7]	88.9 (80/90) [84.3–93.3]	50.0 (10/20) [29.5–70.0]	80.0 (80/100) [75.9–84.0]	75.0 (90/120) [68.2–81.7]	36 ± 15

**Performance of observers with DCR**							
Radiologists (n = 2)	.83	46.7 (14/30) [33.2–53.9]	96.7 (87/90) [92.2–99.1]	82.4 (14/17) [58.5–95.2]	84.5 (87/103) [80.5–86.6]	84.2 (101/120) [77.4–87.8]	32 ± 7
Cardiologists (n = 2)	.89	76.7 (23/30) [61.7–87.0]	92.2 (83/90) [87.2–95.7]	76.7 (23/30) [61.7–87.0]	92.2 (83/90) [87.2–95.7]	88.3 (106/120) [80.8–93.5]	51 ± 23
Radiology residents (n = 2)	.90	86.7 (26/30) [72.2–95.2]	91.1 (82/90) [86.3–93.9]	76.5 (26/34) [63.7–84.0]	95.3 (82/86) [90.3–98.3]	90.0 (108/120) [82.8–94.2]	50 ± 23

**P value (without DCR vs with DCR)**							
Radiologists (n = 2)	.0002	.052	.033	-	-	.0039	.0009
Cardiologists (n = 2)	.0028	.046	.0046	-	-	.0006	< .0001
Radiology residents (n = 2)	.011	.0002	.59	-	-	.0015	< .0001

**Table 5:** Subgroup analysis of observer performance according to the observer’s expertise and experience in the supine position.

	AUC	Sensitivity (%)	Specificity (%)	PPV (%)	NPV (%)	Accuracy (%)	Reading time (s)
**Performance of observers without DCR**							
Radiologists (n = 2)	.61	33.3 (10/30) [19.2–48.9]	80.0 (72/90) [75.3–85.2]	35.7 (10/28) [20.6–52.4]	78.3 (72/92) [73.7–83.3]	68.3 (82/120) [61.3–76.1]	30 ± 9
Cardiologists (n = 2)	.57	40.0 (12/30) [24.6–56.7]	64.4 (58/90) [59.3–70.0]	27.3 (12/44) [16.8–38.7]	76.3 (58/76) [70.2–82.9]	58.3 (70/120) [50.6–66.7]	39 ± 13
Radiology residents (n = 2)	.69	26.7 (8/30) [14.3–38.9]	91.1 (82/90) [87.0–95.2]	50.0 (8/16) [26.8–72.9]	78.8 (82/104) [75.3–82.4]	75.0 (90/120) [68.8–81.1]	45 ± 17

**Performance of observers with DCR**							
Radiologists (n = 2)	.81	53.3 (16/30) [40.8–56.5]	98.9 (89/90) [94.7–99.9]	94.1 (16/17) [71.9–99.7]	86.4 (89/103) [82.7–87.3]	87.5 (105/120) [81.2–89.1]	35 ± 7
Cardiologists (n = 2)	.83	76.7 (23/30) [60.7–88.2]	85.6 (77/90) [80.2–89.4]	63.9 (23/36) [50.6–73.5]	91.7 (77/84) [86.0–95.8]	83.3 (100/120) [75.4–89.1]	49 ± 19
Radiology residents (n = 2)	.78	66.7 (20/30) [51.5–77.8]	92.2 (83/90) [87.2–95.9]	74.1 (20/27) [57.2–86.4]	89.2 (83/93) [84.3–92.8]	85.8 (103/120) [78.2–91.4]	51 ± 16

**P value (without DCR vs with DCR)**							
Radiologists (n = 2)	.013	.11	< .0001	-	-	< .0001	< .0001
Cardiologists (n = 2)	.0024	.012	.0003	-	-	< .0001	< .0001
Radiology residents (n = 2)	.28	.0047	.78	-	-	.020	.0003

### 3.5 Reading time

The reading time of CR with DCR was slightly but significantly longer than that of CR alone in both standing (45 ± 21 s vs. 32 ± 13 s, *P* < 0.0001) (Table 2) and supine (45 ± 16 s vs. 38 ± 15 s, *P* < 0.0001) (Table 3) positions.

In the interpretation of CR with DCR, the reading time of the radiologists (standing, 32 ± 7 s; supine, 35 ± 7 s) were significantly shorter than those of the cardiologists (standing, 51 ± 23 s, *P* < 0.0001; supine, 49 ± 19 s, *P* < 0.0001) and radiology residents (standing 50 ± 23 s, *P* < 0.0001; supine 51 ± 16 s, *P* < 0.0001) (Table 4, Table 5). The difference between the cardiologists and radiology residents was non-significant (standing, *P* = 0.74; supine, *P* = 0.37).

## 4 Discussion

Although DCR may be a promising tool for detecting PE, its diagnostic performance has not yet been investigated. We demonstrated that 1) the pooled observer diagnostic performances of DCR with CR were high, with accuracies exceeding 85 % and AUCs of 0.8 or higher observed in both standing and supine positions; 2) the addition of DCR increased the diagnostic ability and confidence for PE detection, irrespective of the observers' expertise and experience; and 3) the diagnostic performance of CR with DCR was similar in the standing and supine positions.

Incorporating DCR with CR demonstrated moderate sensitivity, high specificity, and high accuracy in detecting PE, all of which were significantly higher than those achieved with CR alone. In a previous study, CR without DCR had a high specificity of > 75 % and an unsatisfactory sensitivity [6]. The addition of DCR to CR significantly improves both sensitivity and specificity. In particular, CR with DCR has an excellent specificity of > 90 %, and a sensitivity of approximately 70 % is clinically applicable. The entrance surface doses for the DCR were 1.0 ± 0.2 mGy and 1.6 ± 0.5 mGy in the standing and supine positions, respectively, which are lower than the sum of dose limits recommended by the International Atomic Energy Agency for posteroanterior (0.4 mGy) and lateral (1.5 mGy) projections [7], [8]. Compared with other imaging modalities, the radiation dose of perfusion DCR (0.2 mSv) is one-tenth that for lung V/Q scanning (2 mSv) and one-twentieth that for standard CECT of the chest (4–6 mSv) [13]. DCR is readily available with almost no contraindications. Additionally, DCR requires only 7 s for imaging and 1 min for postprocessing. The initial installation cost of DCR is estimated to be lower than that of a general CT machine. The operational cost of DCR is also less than that of CECT, as the latter requires additional expenses for contrast agents used in each examination. Owing to its high diagnostic accuracy, CECT is a highly recommended non-invasive modality for patients with suspected PE. Therefore, it should be prioritized in clinical situations where its use is not limited. However, the use of contrast media and high doses of radiation may occasionally be problematic in patients with allergies to contrast media, impaired renal function, or radiosensitivity, such as pregnant women. V/Q scintigraphy, which has almost no contraindications, is a suitable alternative for such patients. However, it is not readily available in all centers because of its high specialty and large size [21]. The combination of the D-dimer test and clinical pretest probability for acute PE, which can be performed even in clinical situations where CECT and scintigraphy are unavailable, has an excellent sensitivity of almost 100 % but insufficient specificity [22], [23], [24], [25]. The high specificity of DCR may complement and strengthen its diagnostic power for PE. This may be helpful for the early initiation of anticoagulation therapy without the use of CECT. The simplicity of DCR has led to the recent development of a portable system [26] and suggests the potential for its installation in trucks in the near future. This would provide an opportunity to diagnose PE among residents in remote areas without access to CT scans.

The AUC of CR with DCR in the supine position was slightly lower than that in the standing position; however, the difference was not statistically significant. Furthermore, both positions demonstrated similar diagnostic accuracies, surpassing 85 %. Typically, chest radiography is conducted with patients in the standing position, although it may be performed in the supine position for individuals experiencing dyspneic or hemodynamic instability. The comparable diagnostic abilities observed in the supine and standing positions may be beneficial in emergencies.

Radiologists, cardiologists, and radiology residents demonstrated similar diagnostic performances when utilizing CR with DCR in the supine position for the detection of PE. However, in the standing position, the radiologists exhibited the lowest AUC value. This might be because all observers, including radiologists, were unfamiliar with DCR images owing to the novelty of DCR. Interestingly, the radiologists exhibited significantly shorter reading times compared to cardiologists and radiology residents. Considering that DCR is a novel imaging method, careful reading with sufficient time after adequate training is encouraged for image interpretation. Further research in larger cohorts is required to validate and investigate whether the diagnostic performance of DCR is influenced by observer expertise and experience.

DCR was recently reported to have high diagnostic efficacy for detecting chronic thromboembolism in patients with pulmonary hypertension, comparable to that of the V/Q scan [13]. Compared with this previous study, the diagnostic accuracy of DCR in our study was similar; however, the sensitivity was lower. The main difference between these studies was the comparative modality of DCR. We used CECT as the reference because it is generally prioritized when acute PE is suspected. Similar to the V/Q scan, DCR evaluates lung perfusion defects due to obstruction of the pulmonary arteries, whereas CECT directly detects thrombi in the pulmonary arteries. Occasionally, pulmonary thrombosis may demonstrate almost normal or slightly decreased lung perfusion when a clot exists but does not obstruct pulmonary flow [27], [28], [29]. In addition, small perfusion defects may go undetected due to the “shine-through masking paradigm,” which occurs due to the superposition of abnormal areas of perfusion over regions with normal perfusion in DCR, same as planar scintigraphy [13], [30]. This could explain the low sensitivity of the DCR for PE in our study. Moreover, we conducted individual interpretations instead of a consensus reading to put the study closer to a clinical situation. A consensus reading has been previously reported to improve the diagnostic performance of DCR [13]. Additionally, CTEPH is a type of pulmonary hypertension. Pulmonary vascular resistance begins to rise after 50 % of the vascular beds are damaged [31], meaning that most patients with CTEPH have extensive pulmonary perfusion defects in their lungs, making detection by DCR both possible and relatively straightforward. In contrast, acute PE is sometimes discovered incidentally during screening CT for chest discomfort or during follow-up CT for cancer without obvious PE-related symptoms. In such cases, the blood clots may be small, and patients may not have large perfusion defects, making detection by DCR more difficult and potentially lowering its sensitivity. With further technological advancements, such as the integration of AI technology, DCR could become a more reliable tool for diagnosing PE.

A recent study investigated the diagnostic accuracy of fluoroscopic video analysis for detecting PE—a method similar to, but distinct from, the reference subtraction analysis used in our study. They reported very high sensitivity, relatively low specificity, and lower overall accuracy compared to our results [32]. As previously mentioned, given the unavoidable limitations of DCR in detecting acute PE, a slightly lower sensitivity is reasonable and aligns with our findings. Additionally, in the aforementioned study, PE diagnosis was based on a comparison of perfusion and respiratory air-filling images. Consequently, nearly half of the non-PE patients (13 out of 27) were incorrectly diagnosed with PE, leading to overestimation and reduced specificity. The higher specificity and diagnostic accuracy in our study may further validate the evaluation criteria we employed, which combine CR and DCR to prevent overdiagnosis and improve diagnostic performance. Although these differences may partly reflect variations in the analysis software used, they warrant further investigation in larger populations in future studies.

This study had some limitations. First, the sample size was small, and the study design was retrospective. which may limit the generalizability of the findings and the ability to establish causality. Second, the interval between the DCR and CT was the highest at 70 h. The possibility of additional thrombosis or thrombolysis cannot be completely ruled out, particularly in patients with PE. Third, the prevalence of PE within our cohort may be higher than that of contemporary CT practice when the disease is suspected clinically. As these limitations are mainly due to the retrospective nature of our study, prospective studies with larger sample sizes are needed to confirm these results in a clinical setting. Fourth, we used both CR and DCR in the interpretation of DCR to avoid overdiagnosis caused by artifacts from lung lesions. However, defects behind lung lesions could be overlooked, which might have led to reduced sensitivity. Lastly, although radiation exposure from DCR is lower than that of other modalities, it is important to keep in mind that it is not entirely free of radiation. Careful consideration is especially needed for radiosensitive patients.

## 5 Conclusions

DCR demonstrated moderate sensitivity, high specificity, and high accuracy in detecting PE. Additionally, we observed a significant enhancement in the diagnostic accuracy for PE by incorporating DCR alongside CR, irrespective of the scan position, observer expertise, and observer experience. These findings highlight the potential value of DCR as a valuable diagnostic tool for PE in various clinical scenarios. DCR can be beneficial in situations where the use of CECT is restricted, such as in local clinics or small hospitals, or when dealing with patients who have contraindications to contrast media (e.g., due to allergy or renal failure) or pregnant women who need to avoid radiation exposure. However, further improvements on its diagnostic performance or the combination of DCR with other diagnostic tests are necessary for its routine clinical use.

## CRediT authorship contribution statement

**Shunsuke Nishimura:** Writing – review & editing, Resources, Investigation, Data curation. **Hiroyuki Toyoda:** Writing – review & editing, Resources, Investigation, Data curation. **Shohei Moriyama:** Writing – review & editing, Resources, Investigation, Data curation. **Masateru Kawakubo:** Writing – review & editing, Methodology. **Koji Sagiyama:** Writing – review & editing, Resources, Data curation. **Takuya Hino:** Writing – review & editing, Resources, Investigation, Data curation. **Megumi Ikeda:** Writing – review & editing, Resources, Investigation, Data curation. **Noritsugu Matsutani:** Writing – review & editing, Software. **Yuzo Yamasaki:** Writing – review & editing, Writing – original draft, Visualization, Validation, Resources, Methodology, Funding acquisition, Formal analysis, Data curation, Conceptualization. **Hidetake Yabuuchi:** Writing – review & editing, Resources, Data curation. **Kazuya Hosokawa:** Writing – review & editing, Data curation, Conceptualization. **Kousei Ishigami:** Writing – review & editing, Supervision, Project administration. **Takeshi Kamitani:** Writing – review & editing, Resources, Investigation, Data curation. **Kohtaro Abe:** Writing – review & editing, Resources, Data curation.

## Ethical statement

This diagnostic study was approved by the institutional review board of Kyushu University Hospital (No. 2022-180), and the requirement for informed consent was waived owing to the retrospective nature of the study.

This study does not include animal experiment. We use only demographic and image data.

Thus, we state that this study is based on the declaration of Helsinki.

## Declaration of Competing Interest

The authors declare the following financial interests/personal relationships which may be considered as potential competing interests: Yuzo Yamasaki reports financial support was provided by Japan Society for the Promotion of Science. Yuzo Yamasaki reports financial support was provided by Konica Minolta Inc. Kazuya Hosokawa reports financial support was provided by Konica Minolta Inc. Takeshi Kamitani reports financial support was provided by Konica Minolta Inc. Kohtaro Abe reports financial support was provided by Konica Minolta Inc. Koji Sagiyama reports financial support was provided by Konica Minolta Inc. Kousei Ishigami reports financial support was provided by Konica Minolta Inc. Yuzo Yamasaki reports financial support was provided by Konica Minolta Science and Technology Foundation. Noritsugu Matsutani reports a relationship with Konica
