# Supplementary material for: Diagnostic accuracy and added value of dynamic chest radiography in detecting pulmonary embolism: A retrospective study
Source: Eur J Radiol Open. 2024 Oct 5;13:100602. doi: 10.1016/j.ejro.2024.100602 (PMC11490836; doi:10.1016/j.ejro.2024.100602)
Supplement: Supplementary file 1 — Supplementary material [file mmc1.docx]

**Supplementary Table and Video Legends**

**Supplementary Table 1**

Scan parameters of dynamic chest radiography

| Position |  | | |
| --- | --- | --- | --- |
|  | BMI [kg/m^2^] | kV | mAs |
| Standing  SID: 2 m  Additional filter: 0.2 mm Cu | BMI < 17 | 85 | 0.64 |
|  | 17 ≤ BMI < 20 | 100 | 0.4 |
|  | 20 ≤ BMI < 23 | 100 | 0.504 |
|  | 23 ≤ BMI < 26 | 100 | 0.568 |
|  | 26 ≤ BMI < 29 | 110 | 0.55 |
|  | 29 ≤ BMI < 32 | 110 | 0.625 |
|  | 32 ≤ BMI | 110 | 0.9 |
| Supine  SID: 1.5 m  Additional filter: 0.3 mm Cu | BMI < 17 | 85 | 0.55 |
|  | 17 ≤ BMI < 20 | 95 | 0.45 |
|  | 20 ≤ BMI < 23 | 95 | 0.625 |
|  | 23 ≤ BMI < 26 | 95 | 0.8 |
|  | 26 ≤ BMI < 29 | 95 | 1 |
|  | 29 ≤ BMI < 32 | 95 | 1.125 |
|  | 32 ≤ BMI | 95 | 1.25 |

BMI, body mass index; SID, source-to-image distance

**Supplementary Table 2**

Observer performances of dynamic chest radiography for the detection of pulmonary embolism in standing and supine positions

|  | AUC | Sensitivity (%) | Specificity (%) | Accuracy (%) |
| --- | --- | --- | --- | --- |
| Overall | | | | |
| Standing position | .85 | 70.0 [62.2−76.3] | 93.3 [90.7−95.4] | 87.5 [83.6−90.6] |
| Supine position | .80 | 65.6 [57.5−72.2] | 92.2 [89.5−94.5] | 85.6 [81.5−88.9] |
| Radiologists | | | | |
| Standing position | .83 | 46.7 [33.2−53.9] | 96.7 [92.2−99.1] | 84.2 [77.4−87.8] |
| Supine position | .81 | 53.3 [40.8−56.5] | 98.9 [94.7−99.9] | 87.5 [81.2−89.1] |
| Cardiologists | | | | |
| Standing position | .89 | 76.7 [61.7−87.0] | 92.2 [87.2−95.7] | 88.3 [80.8−93.5] |
| Supine position | .83 | 76.7 [60.7−88.2] | 85.6 [80.2−89.4] | 83.3 [75.4−89.1] |
| Radiology residents | | | | |
| Standing position | .90 | 86.7 [72.2−95.2] | 91.1 [86.3−93.9] | 90.0 [82.8−94.2] |
| Supine position | .78 | 66.7 [51.5−77.8] | 92.2 [87.2−95.9] | 85.8 [78.2−91.4] |

AUC, area under the curve

**Supplementary Video legends**

**Supplementary Video 1**

CR image (A) and DCR movie (B) of a 67-year-old woman with acute pulmonary thromboembolism captured in the standing position.

CR, chest radiography; DCR, dynamic chest radiography

**Supplementary Video 2**

CR image (A) and DCR movie (B) of a 50-year-old man without pulmonary embolism captured in the supine position.

CR, chest radiography; DCR, dynamic chest radiography
